# A New Survey about Dual-Acting PARP/COX-2 Inhibitors as Cancer Chemopreventive Agents

**DOI:** 10.5812/ijpr-169140

**Published:** 2026-05-26

**Authors:** Mohammad Ismail Mahboubi Rabbani, Ali Aliabadi, Leila Bayat, Mahdokht Jafari, Amir Hossein Bagheri, Sarah Shateri, Rahil Hakimimofrad, Maryam Bayanati, Afshin Zarghi

**Affiliations:** 1 Department of Pharmaceutical Chemistry, School of Pharmacy and Pharmaceutical Sciences, Islamic Azad University, Tehran, Iran; 2 School of Pharmacy, Shahid Beheshti University of Medical Sciences, Tehran, Iran; 3 Phytochemistry Research Center, Shahid Beheshti University of Medical Sciences, Tehran, Iran; 4 Department of Medicinal Chemistry, School of Pharmacy, Shahid Beheshti University of Medical Sciences, Tehran, Iran

**Keywords:** PARP Inhibitor, The Cancer Genome Atlas, COX-2 Enzyme, Dual Inhibitors

## Abstract

**Context:**

Poly(ADP-ribose) polymerase (PARP) is a key enzyme involved in DNA replication. Studies have shown that this enzyme plays a pivotal role in apoptosis and DNA damage repair. Excessive PARP activity, particularly that of PARP-1, may also lead to uncontrolled cellular growth and proliferation, thereby predisposing to tumor development. Similar effects have been observed with the overexpression of cyclooxygenase-2 (COX-2) in cancer development. In this review, our primary objective is to provide a concise and comprehensive structure–activity relationship to guide the design of compounds with dual inhibitory activity against PARP and COX-2. On the basis of this relationship, this review aims to support the development of novel compounds with potent inhibitory activity against PARP-1, as well as the design of dual PARP-1/COX-2 inhibitors.

**Evidence Acquisition:**

A comprehensive literature search was conducted to identify relevant studies on the design, synthesis, and biological evaluation of novel PARP and PARP-1 inhibitors, as well as dual-acting PARP-1/COX-2 inhibitors. Electronic databases, including Scopus, ScienceDirect, and Google Scholar, were systematically searched. Research articles published in English up to the most recent available date were considered. Studies were screened for relevance, clarity, and compliance with recognized molecular design guidelines. The collected information was analyzed and synthesized to provide an overview of the current state of PARP-1/COX-2 inhibitor synthesis approaches and design trends.

**Results:**

Based on the review findings, the use of PARP-1/COX-2 dual inhibitors may be considered a viable therapeutic approach for targeting two interconnected pathways involved in cancer development. Dual-acting agents provide an opportunity to enhance anticancer efficacy and reduce resistance to malignancy. Moreover, preclinical studies have demonstrated synergistic effects. Notably, there are substantial similarities between the SAR of single- and dual-acting PARP-1/COX-2 inhibitors. In addition, polycyclic core structures could be used to guide the design of these compounds. Furthermore, these polycyclic structures may exhibit other important antineoplastic properties, such as inhibition of topoisomerase II (TPO-II).

**Conclusions:**

In summary, to provide a more comprehensive review of the design and synthesis of novel chemical compounds as COX-2/PARP-1 dual inhibitors, additional compounds must be developed. Moreover, further clinical development and evaluation are required to improve their pharmacological properties and confirm their safety and efficacy in clinical settings.

## 1. Context

Poly(ADP-ribose) polymerase (PARP) is a member of a protein superfamily that plays important roles in cell death, DNA repair, and genome integrity. PARP-1 is the best recognized of all PARPs and is extensively involved in DNA damage repair, particularly of single-strand breaks (SSBs) (1). Once DNA is damaged, PARP-1 recognizes the breaks and binds to them to become activated. It then catalyzes ADP-ribosylation, which involves the transfer of ADP-ribose units from nicotinamide adenine dinucleotide (NAD) to target proteins, notably itself. This modification facilitates the recruitment of additional DNA repair proteins to the damage site, thereby enhancing repair efficiency (2). In addition to DNA repair, PARP enzymes regulate chromatin remodeling, cell death, and inflammation (3-6). PARPs modify chromatin structure and gene expression by adding ADP-ribose chains to histones. PARP-1 may trigger cell death when DNA damage is irreversible. Moreover, PARP plays an important role in inflammation by initiating immune responses after cellular stress or damage (6, 7). Given that PARP enzymes are critical for genome stability, there is substantial interest in exploiting them as therapeutic targets, particularly in cancer therapy. PARP inhibitors have been developed to treat cancers with defective DNA repair systems, such as those with BRCA1/2 mutations. These drugs inhibit PARP, thereby preventing cancer cells from repairing their DNA. This leads to the accumulation of DNA damage and ultimately cell death. This approach is based on the concept of "synthetic lethality," which states that when two genes (in this case, PARP and BRCA1/2 (7)) are inactivated, the cell dies, whereas if only one gene is inactivated, the cell survives (8). PARPs are essential for cellular survival and repair pathways; however, dysregulation can exacerbate disease processes, particularly in cancer. Inhibition of PARPs has proven to be a viable therapeutic strategy for some malignancies, underscoring their importance in both fundamental cell biology and clinical practice (7).

Cyclooxygenase-2 (COX-2) is another crucial enzyme associated with the production of prostanoids, particularly prostaglandins (PGs), a class of lipid molecules involved in inflammation, pain, fever, and other biological activities (9, 10). COX-2 is one of the two most common cyclooxygenase isoforms; the other is COX-1. Both enzymes catalyze the conversion of arachidonic acid, a fatty acid present in cellular membranes, into prostaglandins, although COX-2 has distinct functions and is regulated differently from COX-1 (11). COX-1 is constitutively expressed in numerous tissues and plays an important role in maintaining normal physiological functions such as platelet aggregation (12, 13). COX-2, in contrast, is primarily induced under inflammatory conditions (14). Accordingly, COX-2 levels increase markedly in response to trauma, viral infection, or swelling (15-17). COX-2 is primarily involved in the inflammatory response. When tissues are injured or infected, inflammatory mediators, including cytokines, growth factors, and other signals, induce COX-2 expression. This results in the production of prostaglandin E2 (PGE2), along with other prostanoids, which promote pain, fever, and inflammatory responses via vasodilation, increased vascular permeability, and sensitization of pain receptors (18-20).

COX-2 is implicated in numerous pathophysiological conditions associated with chronic inflammation, including arthritis, as well as certain forms of cancer. COX-2 is commonly overexpressed in a variety of cancers, including colorectal, breast, and lung cancer (21, 22). The enzyme promotes tumor growth by accelerating cell division, preventing apoptosis, and increasing vasculature.

**Figure 1.**
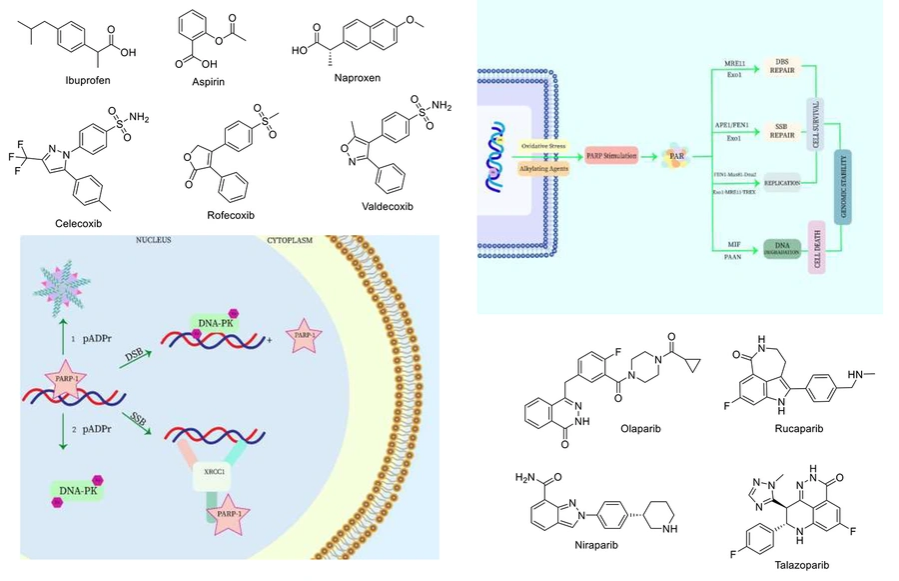
A, chemical structures of well-known classic NSAIDs and celecoxib; B, schematic diagram showing the role of PARP enzymes in cancer development and related signaling pathways. PARP-1 is mainly activated by single-strand DNA breaks and contributes to base excision repair (BER) by adding poly(ADP-ribose) groups to nuclear proteins. Excessive PARP activity promotes genomic instability and resistance to cell death, thereby supporting tumor initiation and progression. PARP-mediated signaling also regulates transcription factors such as NF-κB and HIF-1α, which promote inflammation, angiogenesis, and metabolic alterations in the tumor microenvironment. Dysregulation of PARP activity, particularly in the context of impaired homologous recombination repair, such as BRCA mutations, facilitates oncogenic transformation and supports the targeting of PARP in cancer therapy. C, chemical structures of selected well-known PARP-1 inhibitors.

NSAIDs (nonsteroidal anti-inflammatory drugs) are commonly used to reduce inflammation and discomfort by inhibiting COX. Ibuprofen and aspirin (Figure 1) are two conventional NSAIDs that inhibit both COX-1 and COX-2. However, because COX-1 protects the gastric mucosa, this broad inhibition may result in adverse effects such as inflammation, ulceration, and hemorrhage. To mitigate these adverse reactions, COX-2-selective inhibitors, or coxibs, such as celecoxib (Figure 1A), were developed (15).

These medications selectively target COX-2 to alleviate pain and inflammation while preserving the beneficial effects of COX-1 on the gastrointestinal lining and blood coagulation. However, concerns remain regarding the cardiovascular risks associated with long-term COX-2 inhibition, as it may interfere with normal platelet aggregation and increase the risk of cardiac events or stroke (23).

In this review, our main goal is to provide a concise and comprehensive structure-activity relationship for the design of compounds with dual inhibitory activity against PARP and COX-2. Based on this relationship, this review aims to support the development of novel compounds with inhibitory potency against PARP-1, as well as the design of dual PARP-1/COX-2 inhibitors.

## 2. Evidence Acquisition

A comprehensive literature search was conducted to identify studies relevant to the design, synthesis, and biological evaluation of novel PARP and PARP-1 inhibitors, as well as dual-acting PARP-1/COX-2 inhibitors. Electronic databases, including Scopus, ScienceDirect, and Google Scholar, were systematically searched. Research articles published in English up to the most recent available date were considered. Studies were screened for relevance, clarity, and compliance with established molecular design guidelines. The collected information was analyzed and synthesized to provide an overview of the current state of synthesis approaches and design trends for PARP-1/COX-2 inhibitors.

## 3. Results

### 3.1. PARP-1 Roles in the Pathophysiology of Cancer

Cancer is the world’s second-largest cause of death and was responsible for an estimated 9.6 million deaths in 2018. As discussed above, PARPs are enzymes involved in multiple processes in cancer development, including DNA repair, gene regulation, chromatin remodeling, and apoptosis. The first characterized and best-known member of the PARP family is PARP-1, which is a major protein involved in DNA single-strand break repair in the base excision repair (BER) pathway (24).

Excessive or dysregulated PARP activity can lead to erroneous DNA-break repair, the accumulation of mutations, and chromosomal instability, which is a hallmark of cancer and promotes tumor growth (25). Excessive PARP activity may enable these cells to survive extensive DNA damage by repairing lesions sufficiently to avoid cell death, but it does not prevent mutation accumulation. PARP-1 modifies histones and transcription factors through PARylation, thereby altering chromatin architecture. This may activate oncogenes or silence tumor suppressor genes, facilitating cancer growth and metastasis. PARP-1 modulates the activity of transcription factors such as NF-κB (26), which regulate pro-inflammatory cytokines. Chronic inflammation driven by PARP-1 signaling creates an environment conducive to tumor promotion, similar to the role of COX-2 as a major physiological enzyme (27). Conversely, the therapeutic importance of PARP inhibition is based on the concept of synthetic lethality. Cancer cells with BRCA1 or BRCA2 mutations have impaired HR repair and therefore rely on PARP-mediated repair pathways for survival (28). In some cancer cells, PARP activity helps them evade apoptosis, enabling survival under stressful conditions (Figure 1B) (27). Hence, suppressing the PARP pathway is a promising strategy to combat intractable forms of neoplasia. One of the most extensively studied PARP1 inhibitors is Olaparib (29) (Figure 1C). Notably, the BER pathway uses PARP to repair breaks in single-stranded DNA. In normal cells with intact homologous recombination (HR) repair mechanisms, particularly functional BRCA1/2, PARP inhibition typically causes DNA damage accumulation that can still be repaired by HR. Therefore, normal cells generally tolerate PARP inhibitors, contributing to their favorable therapeutic index (30).

### 3.2. COX-2 Roles in the Pathophysiology of Cancer

**Figure 2.**
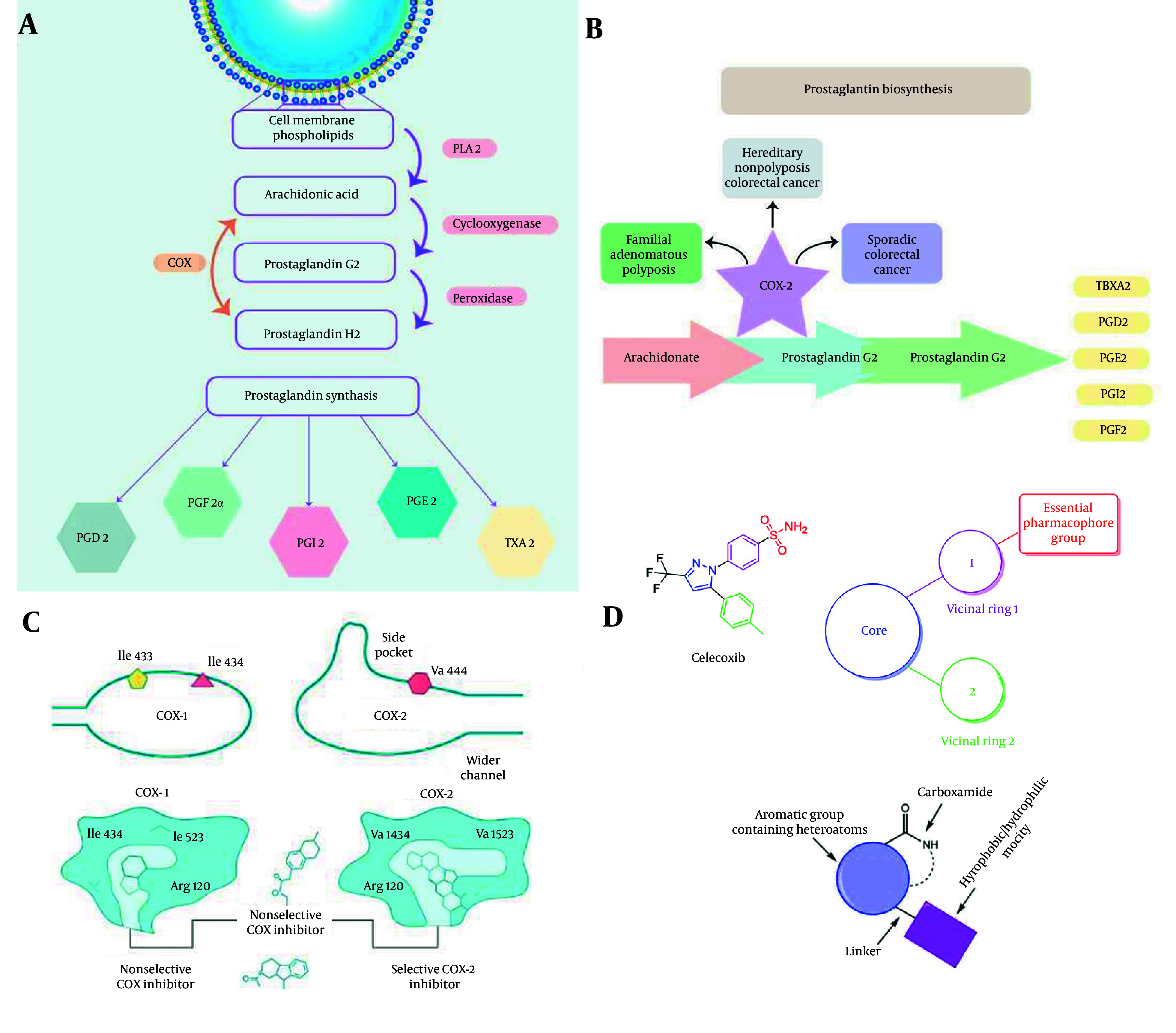
A, Schematic diagram showing the role of COX enzymes in prostaglandin biosynthesis. Phospholipase A2 hydrolyzes membrane phospholipids to release arachidonic acid. COX-1 and COX-2 then convert arachidonic acid into the unstable intermediate prostaglandin G2 (PGG2), followed by prostaglandin H2 (PGH2). PGH2 serves as the universal substrate for downstream synthases, yielding bioactive prostanoids such as prostaglandins (PGE2, PGD2, and PGF2α), prostacyclin (PGI2), and thromboxane A2 (TXA2). These mediators are involved in several physiological and pathological processes, including inflammation, vascular homeostasis, and platelet aggregation. B, Proposed role of COX enzymes in cancer initiation and progression. COX-1 and COX-2 convert arachidonic acid into PGH2, which is then converted into bioactive prostanoids such as PGE2. Elevated PGE2 levels promote tumorigenic processes, including increased cell proliferation, angiogenesis, evasion of apoptosis, immune suppression, and metastasis. COX-2 overexpression has been consistently associated with chronic inflammation, tumorigenesis, and unfavorable clinical outcomes in various malignancies. C and D, SAR of COX-2 and PARP-1 inhibitors. Key pharmacophoric features are illustrated, including aromatic heterocycles that facilitate hydrogen bonding at catalytic sites, hydrophobic substituents that enhance binding affinity, and electron-withdrawing groups that improve selectivity. In PARP-1 inhibitors, planar heteroaromatic cores mimic the nicotinamide moiety of NAD⁺, facilitating competitive interaction with the catalytic domain. In COX-2 inhibitors, sulfonamide or methylsulfonyl groups occupy the secondary pocket, enhancing selectivity for COX-2 over COX-1. The figure demonstrates how minor structural modifications can markedly affect biological activity, potency, and therapeutic efficacy.

COX-2 plays a critical role in the pathophysiology of cancer. COX-2 is usually not detectable under normal physiological conditions, but it becomes highly induced in response to pro-inflammatory signals, growth factors, and oncogenes (20). This differs from its constitutive isoform, COX-1. COX-2 promotes prostaglandin E2 (PGE2) production in the tumor microenvironment (Figure 2A), which supports tumor growth by accelerating cell proliferation, reducing genomic stability, and increasing resistance to apoptosis (31). COX-2 also increases levels of angiogenic factors such as vascular endothelial growth factor (VEGF), thereby promoting neovascularization, which is essential for tumor growth and metastasis (Figure 2B) (32).

Beyond supporting tumor expansion, COX-2 contributes to invasion and metastatic spread by stimulating epithelial–mesenchymal transition (EMT), enhancing cell motility, and increasing the expression of matrix metalloproteinases (MMPs) (34). It also promotes immune evasion by inhibiting dendritic cell and cytotoxic T-cell function and facilitating the expansion of regulatory T cells (Tregs) (35). Sustained COX-2 activity can drive chronic inflammation, which has been linked to the initiation and progression of many cancers, including colorectal, gastric, and pancreatic cancers (36). Given its multiple roles, COX-2 is an attractive target for chemoprevention and therapy. For example, selective COX-2 inhibitors such as celecoxib have demonstrated efficacy in familial adenomatous polyposis (FAP) and are being investigated in other cancers (Figure 2B) (37).

### 3.3. Structure-Activity Relationship of PARP-1 and COX-2 Inhibitors

The structure-activity relationship (SAR) of PARP inhibitors is determined by their ability to emulate nicotinamide and occupy the NAD^+^ binding site of PARP (38). Core scaffolds engage critical catalytic residues, whereas specific substituents modulate potency, PARP-DNA trapping efficacy, selectivity across PARP isoforms, and pharmacokinetic properties. Minor structural modifications can markedly influence cytotoxicity and therapeutic efficacy, underscoring the need to achieve an optimal balance between drug-like properties and enzyme inhibition (Figure 2D) (39).

The SAR of COX-2 inhibitors is primarily dictated by their ability to selectively bind to the hydrophobic accessory site of the COX-2 enzyme while avoiding COX-1 interaction (Figure 2C) (23). Key features include a centrally positioned aromatic or heteroaromatic scaffold, a sulfonamide or analogous polar “selectivity” isosteric group that occupies the COX-2 side pocket, and hydrophobic substituents that enhance enzyme binding and efficacy (23). Modifications to these groups influence COX-2 selectivity, the drug’s anti-inflammatory efficacy, pharmacokinetics, and safety profile, thereby aiding the development of anti-inflammatory agents with reduced gastric mucosal toxicity (Figure 2D).

In earlier papers, we described the SAR of selective COX-2 inhibitors (16, 40) as well as dual-acting inhibitors of this enzyme with other targets, such as carbonic anhydrase (41), aromatase (42), and TNF-α (23). In our previously published manuscript (41), we particularly emphasized the results of a major study conducted by one of the most prestigious research teams active in synthesizing carbonic anhydrase inhibitors (43). Furthermore, our research team has generated several unique chemical structures as selective COX-2 inhibitors (44-47). In the present review, a concise explanation of the minimum chemical structure requirements for designing selective COX-2 and PARP-1 inhibitors, as well as dual COX-2/PARP-1 inhibitors, is provided. In this regard, we discuss some of the most recently published chemical structures as selective COX-2 inhibitors, PARP-1 inhibitors, and dual-acting COX-2/PARP-1 inhibitors.

### 3.4. Review of the Chemical Structures

#### 3.4.1. Novel PARP Inhibitor Structures

**Figure 3.**
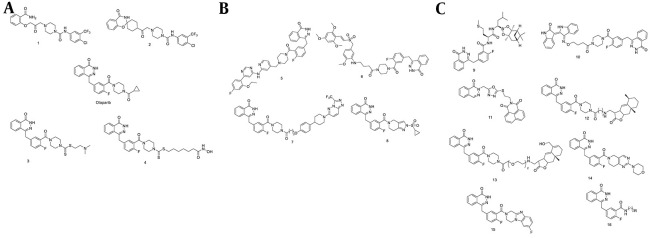
A, Spirobenzoxazinone-based and phthalazinone-based PARP-1 inhibitors 1 - 4 and olaparib. B, Chemical structures of phthalazinone analogs 5 - 8. C, Chemical structures of phthalazinone analogs 9 - 16.

First, we reviewed novel structures developed by researchers in 2025. In a study performed by Kadry et al., a group of spirobenzoxazinone-based derivatives was designed and evaluated as novel PARP-1 inhibitors. Among the newly developed analogs, compounds 1 and 2 (Figure 3A) showed notable antiproliferative effects against H1299 and FaDu cancer cells while maintaining low toxicity in normal fibroblasts. Western blot analysis confirmed effective PARP-1 cleavage, and combination with doxorubicin revealed synergistic anticancer activity. Molecular docking demonstrated binding comparable to Olaparib (48).

A study reported the design and synthesis of novel phthalazinone-based PARP-1 inhibitors and dual PARP-1/HDAC-1 inhibitors. Compound 3 (Figure 3A) showed exceptional PARP-1 inhibition with IC_50_ values below 0.2 nM and exhibited potent antiproliferative effects and G1 phase arrest in breast cancer cells. Among dual inhibitors, 4 demonstrated strong dual-target inhibition and significant anticancer activity, with 4 inducing G2 phase arrest and apoptosis in HCT-116 cells (49).

In a study carried out by Huang et al., dual CDK9/PARP inhibitors with potent anticancer properties were developed. Among the newly developed analogs, compound 5 (Figure 3B) exhibited strong nanomolar inhibitory activity against both targets and broad antiproliferative effects. Compound 5 induced apoptosis, cell-cycle arrest, and migration inhibition in MDA-MB-231 cells (50).

Most TNBC patients lack BRCA mutations and cannot benefit from PARP inhibitors. To address this, dual-target molecules combining the pharmacophores of Olaparib and Rigosertib were developed. Among these, compound 6 (Figure 3B) exhibited significant PARP-1 inhibition and approximately 34-fold enhanced activity compared to Olaparib in BRCA wild-type TNBC cells (MDA-MB-231). The new molecules induced apoptosis through multiple mechanisms and more effectively inhibited tumor growth than Olaparib alone or in combination with other drugs, without causing major systemic toxicity (51).

In a research study led by Zhang et al., novel dual inhibitors targeting androgen receptor variants (AR/AR-Vs) and PARP1 were developed to treat castration-resistant prostate cancer (CRPC). The lead compound 7 (Figure 3B) showed potent inhibition of AR signaling and PARP1, with micromolar IC_50_ values in CRPC cell lines. Compound 7 effectively suppressed proliferation and migration and induced apoptosis in vitro (51).

A study reports the design and synthesis of novel piperidine-based benzamide derivatives as PARP-1 inhibitors. Compound 8 (Figure 3B) showed strong antiproliferative activity against MDA-MB-436 breast cancer cells and was highly effective at inhibiting PARP-1. Mechanistic investigations demonstrated that these compounds inhibited colony formation and migration of HCT116 cells while inducing apoptosis through modulation of Bax, Caspase-3, and Bcl-2. A mouse xenograft model showed that 15d has therapeutic potential, making it a promising candidate for an anticancer drug (52).

Researchers generated dual PARP-1/proteasome inhibitors by combining Olaparib and Ixazomib. Both compounds exhibited significant antiproliferative effects and synergistic activity in PARP-1 inhibitor-resistant cells by downregulating BRCA1 and RAD51, consequently inhibiting homologous recombination repair (HRR). Compound 9 (Figure 3C) demonstrated superior induction of apoptosis and inhibition of breast cancer cell proliferation compared with the template molecule (53).

A series of bifunctional molecules combining PARP inhibitor pharmacophores with indirubin scaffolds was designed to induce DNA damage and inhibit PARP. Compound 10 (Figure 3C) exhibited superior PARP1 inhibition relative to Olaparib and enhanced antiproliferative effects against HCT-116 cells compared to the combination of Olaparib and indirubin-3′-monoxime. Compound 10 showed low toxicity to normal cells but induced γH2AX accumulation, S-phase cell-cycle arrest, and apoptosis. In vivo, compound 10 showed stronger antitumor activity than positive controls, indicating its potential as a candidate cancer therapy (54).

A new study introduces DiPT-4, a dual inhibitor of PARP1 and TOP1, designed to overcome chemoresistance and reduce toxicity compared with other combination therapies. Compound 11 (Figure 3C) exhibited significant cytotoxicity against various cancers while demonstrating minimal toxicity to normal cells. It induces DNA double-strand breaks, arrests the cell cycle, and causes cell death (55).

Researchers designed hybrid PARP1 inhibitors by merging Olaparib with Alantolactone, creating compounds such as 12 and 13 (Figure 3C). These hybrids showed stronger PARP1 inhibition and improved antiproliferative effects in BRCA1-deficient cancer cells compared with Olaparib. They induced DNA damage, caused G2/M cell-cycle arrest, and triggered apoptosis (56).

A new series of PARP inhibitors containing a bicyclic tetrahydropyridine pyrimidine scaffold was developed to target triple-negative breast cancer (TNBC), which typically resists PARP inhibition despite overexpression of RTKs. These compounds were more effective than Olaparib, particularly in TNBC models. Compound 14 (Figure 3C) showed strong PARP inhibition and reduced EGFR and phosphorylated EGFR levels, indicating efficacy in EGFR-overexpressing TNBC. In addition, combining these inhibitors with Adriamycin resulted in a significant synergistic antitumor effect (57).

Two new series of chemicals with inhibitory capability against PARP-1 were identified as a novel group of PARP-1 inhibitors. Among the newly developed analogs, compound 15 displayed impressive results in PARP-1 enzyme inhibition with an IC_50_ value of 0.51 nM and antiproliferative activity against HCT116 and HCC1937 cell lines (58) (Figure 3C). In another paper, a novel series of Olaparib derivatives was introduced as lead compounds with the general molecular formula 16 (Figure 3C). The ability of the compounds to inhibit PARP-1 enzyme activity was evaluated using an intracellular PARylation assay. The findings showed that the inhibitory capabilities of the newly designed analogs corresponded to the nature of the substituent and the total length of the alkyl chain. It was also demonstrated that the potent chemicals against PARP-1 possess strong antiproliferative activity against Capan-1 cells (59).

**Figure 4.**
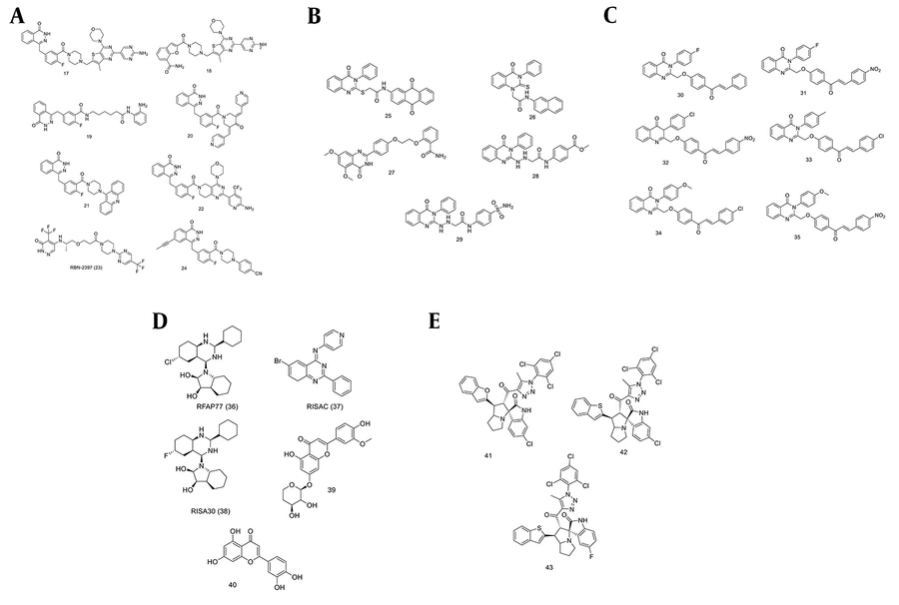
A, PARP/PI3K dual inhibitors 17 and 18, dual PARP-1/HDAC-1 inhibitor 19, dual PARP-1/HSP-90 inhibitor 20, phthalazinone acridine derivative 21, dual PARP/PI3K inhibitor 22, and selective PARP7 inhibitors 23 and 24. B, Novel quinazolinone derivatives, including S-alkylated and N-alkylated derivatives 25 and 26, PARP1/BRD4 dual-target small-molecule inhibitor 27, and phthalazinone-core derivatives 28 and 29. C, Chemical structures of quinazolinone-chalcone hybrid molecules 30 - 35. D, Quinazoline derivatives 36 - 38 and luteolin derivatives 39 and 40. E, Spirooxindole-triazole scaffolds 41 - 43.

Recent evidence indicates that simultaneous administration of PARP and PI3K inhibitors may provide unexpected beneficial effects in several malignancies, including those that are BRCA-competent. In one work, a series of PARP/PI3K dual inhibitors was synthesized and evaluated for biological activity. Compounds 17 and 18 (Figure 4A) exhibited excellent inhibitory activity against PARP-1 and promising antiproliferative activity against both BRCA-deficient and BRCA-competent cancer cells. Compounds 17 and 18 also exhibited significantly high in vivo antiproliferative activity in an MDA-MB-468 xenograft simulation (60).

A study explored dual inhibition of PARP-1 and HDAC-1 as a potential cancer treatment strategy by combining pharmacophores from Olaparib (PARP inhibitor) and Chidamide (HDAC inhibitor). Compound 19 (Figure 4A) showed strong dual activity, comparable to the parent drugs. It also exhibited notable anticancer effects in BRCA1/2-proficient K562 and MDA-MB-231 cells (61).

To improve the effectiveness of PARP inhibitors, researchers developed a series of dual PARP-1/HSP90 inhibitors by combining Olaparib with a curcumin-derived HSP90 inhibitor. Compound 20 (Figure 4A) was one such compound; it binds to HSP90 and reduces BRCA1 expression, suggesting greater anticancer efficacy (61).

A series of phthalazinone acridine derivatives was designed as dual inhibitors targeting PARP and topoisomerase II. Most compounds inhibited proliferation across multiple cancer cell lines. All showed Topo II inhibition at 10 μM and strong PARP-1 inhibition. Compound 21 notably induced apoptosis and caused S-phase cell-cycle arrest in HCT116 colorectal cancer cells (62) (Figure 4A).

A study reports the design of dual PARP/PI3K inhibitors by combining pharmacophores from both inhibitor classes. Compound 22 (Figure 4A) exhibited significant inhibitory activity against PARP-1/2 and PI3Kα/δ. It more effectively inhibited the growth of both BRCA-deficient and BRCA-proficient cancer cells. In an MDA-MB-468 xenograft model, compound 22 outperformed the combination of Olaparib and BKM120 in inhibiting tumor growth without noticeable toxicity (63).

A pan-PARP inhibitor was converted into a selective PARP7 inhibitor, KMR-206. Similar to the known inhibitor RBN-2397 (23), compound 24 (KMR-206) (Figure 4A) synergistically enhanced type I interferon (IFN-β) expression when combined with NA-sensor ligands in mouse embryonic fibroblasts and induced IFN-β alone in mouse colon carcinoma cells (64).

Two series of novel quinazolinone derivatives, S-alkylated and N-alkylated, were designed and synthesized as potential PARP-1 inhibitors, using the quinazolinone scaffold as a bioisostere of Olaparib’s phthalazinone core. These compounds were evaluated for cytotoxicity against the MCF-7 breast cancer cell line. The most potent compounds, 25 and 26 (Figure 4B), showed IC_50_ of 11.4 μM and 10.6 μM, respectively, outperforming Doxorubicin. Both compounds inhibited PARP-1 close to Olaparib (65).

A study developed the first dual-target small-molecule inhibitor that simultaneously targets PARP1 and BRD4, two key proteins with a synthetic lethal interaction in breast cancer. Through fragment-based screening and optimization, compound 27 was identified, showing micromolar activity against both targets. It modulated BRD4/PARP1 expression, induced apoptosis, and caused G1 phase cell-cycle arrest in breast cancer cells. In vivo, 27 (Figure 4B) significantly inhibited tumor growth in BRCA1/2 wild-type models (MDA-MB-468, MCF-7) without notable toxicity. These findings highlight dual BRD4/PARP1 inhibition as a promising strategy for treating breast cancer (66). Using a 4-quinazolinone scaffold as a substitute for the phthalazinone core in Olaparib, researchers developed new PARP-1 inhibitors. Both 28 and 29 (Figure 4B) induced G2/M cell-cycle arrest and enhanced apoptosis in MCF-7 breast cancer cells (67). Among them, compound 29 showed potent activity comparable to Olaparib.

In research performed by Madbouly and colleagues, quinazolinone-chalcone hybrid derivatives were synthesized and investigated for cytotoxic activity against human cancer cell lines. Five human cancer cell lines were used, including A549 lung adenocarcinoma, A431 epidermoid carcinoma, HT-1080, MDA-MB-231, and PC-3, as well as healthy AG-01523 cells. Among the newly developed analogs, compounds 30 - 35 (Figure 4C) showed the highest cytotoxic activity against A431 cells (68).

In a computational study, a large set of quinazoline derivatives was developed to identify novel PARP inhibitors targeting breast cancer. Three top candidates, RFAP77 36, RISA30 37, and RISAC 38 (Figure 4D), showed strong docking scores and favorable binding free energies compared to an approved inhibitor. ADMET predictions indicated favorable drug-like properties, while molecular dynamics simulations confirmed the stability and significant conformational changes of the PARP-ligand complexes (69).

In another computational study, luteolin derivatives were also investigated as potential therapeutics for triple-negative breast cancer (TNBC). Using molecular docking and dynamics simulations, compounds 39 and 40 (Figure 4D) showed strong binding affinity and stable interactions with the TNBC protein (70).

In a study, new compounds based on spirooxindole-triazole scaffolds were designed and synthesized for dual inhibition of EGFR and PARP-1. Compounds 41 - 43 (Figure 4E) showed strong cytotoxicity against HepG2 cancer cells, comparable to or better than doxorubicin, while sparing normal THLE-2 cells. They effectively inhibited EGFR and PARP-1, with IC_50_ values close to or better than the reference drugs Erlotinib and Olaparib (71).

**Figure 5.**
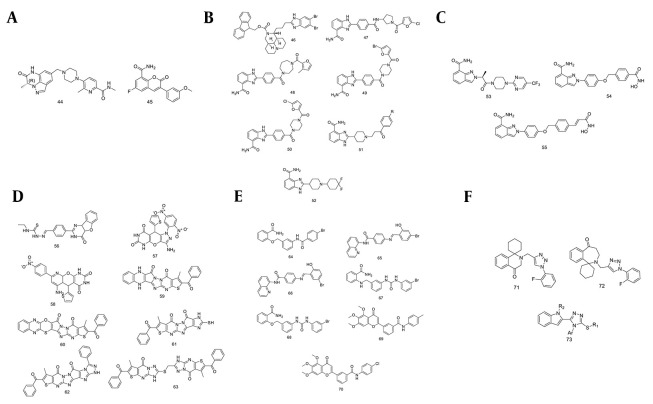
A, Selective PARP1 inhibitor 44 and 8-carbamyl-3-arylcoumarin derivatives 45. B, Benzimidazole scaffold, furan-substituted derivatives 49 and 50, piperidyl benzimidazole carboxamide derivatives 51, and carboxamide derivatives 52. C, Indazole derivatives, carboxamide derivative 53, and PARP-1/HDAC dual-targeting inhibitors 54 and 55. D, PARP inhibitors featuring pyrimidinone scaffold, thiosemicarbazone derivative 56, pyrano derivatives 57 and 58, and thiazine, imidazole, pyrrole, and thienotriazolopyrimidine derivatives 59 - 63. E, Benzamide derivatives, including compounds containing benzamidophenyl and phenylacetamidophenyl scaffolds 64, 4-(benzylideneamino)-N-(quinolin-8-yl)benzamide moiety 65 and 66, urea-based benzamide derivatives 67 and 68, and flavone-based arylamide derivatives 69 and 70. F, Triazole derivatives, Erythrina derivative 71, alkaloid derivative 72, and alkylsulfanyl-triazole derivative 73.

Selective PARP1 inhibitors are being pursued to reduce the hematologic toxicities observed with PARP1/2 inhibitors used in HR-deficient cancers. Compound 44 (Figure 5A), a novel selective PARP1 inhibitor, showed stronger PARP-1-DNA trapping and better anticancer potency than AZD9574. The compound induced tumor regression in BRCA1-mutated models and demonstrated synergistic effects when combined with carboplatin.

In a study, novel 8-carbamyl-3-arylcoumarin derivatives were identified as potent PARP-1 inhibitors based on a natural scaffold. Compound 45 (Figure 5A) showed strong antiproliferative activity against BRCA-mutated cancer cell lines with low micromolar IC50s and effectively inhibited intracellular PARP-1/2 at nanomolar levels (72).

In a study, benzimidazole derivatives were developed as dual inhibitors of TOPOI and PARP-1 to enhance anticancer efficacy. Compound 46 (Figure 5B) showed potent inhibition of both targets, suppressing cancer cell proliferation and migration and inducing DNA damage, G0/G1 cell-cycle arrest, and apoptosis in HGC-27 cells (73).

To elucidate the structure-activity relationship at the ADP-ribose binding location (AD site) of PARP-1 inhibitors, several classes of 2-phenyl-benzimidazole-4-carboxamide analogs incorporating various saturated nitrogen-containing heterocycles as connecting groups were created, synthesized, and assessed for PARP-1 inhibitory activity and proliferation inhibition against the BRCA-1 mutant MDA-MB-436 cell line in vitro. The results indicated that compound 47 (Figure 5B) demonstrated the highest PARP-1 enzyme inhibitory activity, similar to Olaparib, whereas compound 48 (Figure 5B) demonstrated particularly potent antiproliferative activity against MDA-MB-436 cells, akin to Olaparib (74).

In another similar study of PARP-1 inhibitors, inhibitory activity against MDA-MB-436 and MCF-7 cells was assessed using the PARP kit assay; furan derivatives showed greater PARP-1 inhibitory activity than other heterocycles. Among the newly developed analogs, compound 49 (Figure 5B) showed the strongest inhibitory effect on PARP-1, close to that of Olaparib. Differential analysis revealed that compounds 49 and 50 (Figure 5B) were highly effective at inhibiting the proliferation of MDA-MB-436 cells but were inactive against the MCF-7 cell line. This outcome highlights the substantial selectivity and targeting capability of these agents (75). Researchers successfully synthesized a novel series of piperidyl benzimidazole carboxamide derivatives 51 (Figure 5C) and subsequently screened them for PARP-1 inhibitory activity. Several compounds emerged as potent inhibitors with significant anticancer potential. Furthermore, computational simulations were employed, which not only predicted favorable ADME properties but also elucidated the proposed binding modes within the PARP-1 enzyme active site (76).

In a study, two series of benzimidazole carboxamide derivatives containing cyclic amines were synthesized as potential anticancer agents. Many showed strong PARP1/2 inhibitory activity and cytotoxic effects on cancer cell lines. Notably, compound 52 (Figure 5B) demonstrated potent PARP-1 and PARP-2 inhibition, along with selective antitumor activity, good metabolic stability, and an excellent ADME profile (77).

A series of indazole-7-carboxamide derivatives was designed based on RBN-2397. Among them, 53 (Figure 5C) emerged as a potent PARP7 inhibitor with higher selectivity and substantially better oral bioavailability than RBN-2397. In a mouse cancer model, 53 showed strong antitumor activity by activating T-cell immunity in the tumor environment (78).

PARP-1, along with histone deacetylase (HDAC), is a significant candidate for anticancer therapy. PARP-1/HDAC dual-purpose inhibitors were created using benzopyrazole or benzimidazole as core architectures. Compounds 54 and 55 (Figure 5C) were shown to be simultaneous inhibitors of PARP-1 and HDAC6, exhibiting significant antiproliferative activity against six human cancer cell lines. Compounds 54 and 55 may inhibit malignant cell growth more effectively than combination therapy with Olaparib and other cancer chemopreventive medicines. Compound 54 exhibited significant inhibition of migration and anti-angiogenic properties (79).

PARP-1 was targeted using a pyrimidine-4(3H)-one scaffold to design novel inhibitors incorporating thiosemicarbazone derivatives. Among the synthesized compounds, 56 (Figure 5D) showed stronger PARP-1 inhibition and higher selectivity than Olaparib. Notably, compound 56 demonstrated potent activity against PARP-1 and high selectivity over PARP-2 (80).

In a work carried out by Abd El-sattar et al., a new series of pyranopyrimidine-dione derivatives was developed as potential PARP-1 inhibitors. The newly introduced compounds were evaluated for PARP-1 inhibitory activity and antiproliferative activity against HCT116 and MCF-7 cancer cell lines. Among the newly synthesized analogs, compounds 57 and 58 (Figure 5D) showed strong PARP-1 inhibition along with potent cytotoxicity against MCF-7 cancer cells (81).

In an article, the synthesis of new polycyclic aromatic compounds based on the [2,3-d] pyrimidin-4(3H)-one structure was reported. A series of thiazine, imidazole, pyrrole, and thienotriazolopyrimidine derivatives was synthesized and evaluated for antiproliferative activity against four human cancer cell lines: nasopharyngeal CNE2, oral KB, MCF-7, and MGC-803 gastric carcinoma cells. Compounds 59 - 63 (Figure 5D) showed significant cytotoxicity against these human cancer cell lines (82).

A research team led by Zou developed a new series of benzamide derivatives featuring phenylacetamidophenyl and benzamidophenyl moieties as PARP-1 inhibitors with anticancer activity. Among the newly developed analogs, compound 64 (Figure 5E) showed the most potent anticancer activity against HCT116 and DLD-1 colorectal cancer cell lines, with low antiproliferative activity against normal NCM460 colon epithelial cells. Compound 64 also exhibited significant PARP-1 inhibitory activity and suppressed colony formation and migration of HCT116 cells. It was also reported that the aforementioned compound may induce G2/M cell-cycle arrest, DNA strand breakages, mitochondrial membrane potential reduction, and apoptosis (83).

A new class of PARP-1 inhibitors containing the 4-(benzylideneamino)-N-(quinolin-8-yl)benzamide moiety was created in research by Lakshmanan and associates. Compounds 65 and 66 (Figure 5E), two of the recently created analogs, showed docking scores comparable to Olaparib. Enzyme inhibition assays confirmed that these compounds have PARP-1 inhibitory activity similar to Olaparib. Additionally, the compounds showed promising anticancer activity against MCF-7 and MDA-MB-232 cell lines, with 65 and 66 being the most potent (84).

A series of novel urea-based benzamide derivatives was synthesized, and anticancer activity was evaluated against five human cancer cell lines. Compounds 67 and 68 (Figure 5E) showed potent antiproliferative effects on HCT116 cells and strong PARP-1 inhibition. These compounds also inhibited colony formation and cell migration, caused G2/M cell-cycle arrest, and induced apoptosis by modulating apoptotic proteins (85).

A large number of arylamide analogues based on flavones was produced and biologically tested in response to the pressing need to expand the current therapeutic space. To conduct a thorough evaluation, sixty different cancer cell lines were used to assess the efficacy, spectrum, and potency of these drugs. These cell lines represent nine different cancer illnesses with distinct origins. Outperforming masitinib and imatinib, compounds 69 and 70 (Figure 5E) were identified as powerful, general-purpose anticancer medicines. A mechanistic investigation in HT-29 colon cancer cells revealed that compounds 69 and 70 cause cell-cycle arrest (86).

A study designed and synthesized 44 new erythrina derivatives containing a 1,2,3-triazole group as PARP-1 inhibitors. Among them, compound 71 (Figure 5F) showed the strongest antiproliferative effect against A549 lung cancer cells, outperforming the clinical drug rucaparib in both enzyme inhibition and selectivity. Compound 71 induced apoptosis via the mitochondrial pathway by increasing the Bax/Bcl-2 ratio and caspase-3 activation (87).

In a new series of naturally occurring alkaloids featuring a 1,2,3-triazole motif as novel PARP-1 inhibitors, compound 72 (Figure 5F) showed strong activity in suppressing A549 cell proliferation, exceeding that of rucaparib as well as the well-known anticancer compound pemetrexed. Compound 72 notably halted the cell cycle in S phase and subsequently triggered apoptosis in A549 cells, thereby effectively suppressing cell division. Subsequent analytical findings indicated that compound 72 can block cyclin A replication, reduce Bcl-2/Bax activity, trigger caspase-3, and ultimately promote apoptosis in A549 cells (76).

A study used structure-based drug design on a library focusing on triazole-thione and alkylsulfanyl-triazole scaffolds to identify novel PARP-1 inhibitors. Among the newly developed analogs, compound 73 (Figure 5F) demonstrated considerable binding affinity, suppressed PARP-1 activity, and showed promising antiproliferative effects in different cell lines (88).

**Figure 6.**
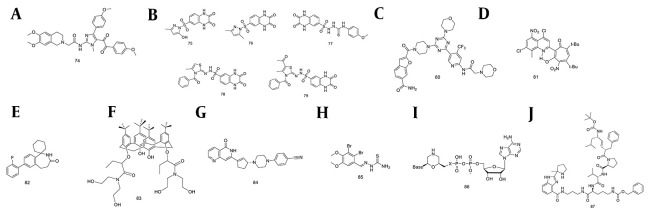
A, 2-Aminoimidazole lissodendrin B derivative 74. B, 2,3-Dioxo-1,2,3,4-tetrahydroquinoxaline derivatives 75 - 79. C, PARP/PI3K dual inhibitor 80. D, Tropolone derivative 81. E, Amide-based PARP-1 inhibitor 82. F, Calixarene carbonyl amide derivative 83. G, Isoquinolinone and naphthyridinone analog 84. H, Bromophenol-thiosemicarbazone hybrid 85. I, Morpholinonucleoside as a potent inhibitor of PARP-1, PARP-2, and PARP-3 86. J, XJB-veliparib, a mitochondria-targeting PARP inhibitor 87.

In a study, 2-aminoimidazole Lissodendrins B derivatives were developed as PARP1 inhibitors. Among them, compound 74 (Figure 6A) showed the strongest inhibition of PARP1 enzymatic activity and effectively suppressed the growth of BRCA1-deficient cancer cells. Compound 74 was found to reduce PARylation, increase DNA double-strand breaks, induce G2/M cell-cycle arrest, and promote apoptosis (89).

In a detailed study, dioxotetrahydroquinoxaline was used as a bioisosteric scaffold for the phthalazinone motif of Olaparib to introduce novel PARP-1 inhibitors. The newly developed analogs 75 - 79 (Figure 6B) were evaluated as antiproliferative agents against MDA-MB-436 cells. Among these ligands, compound 76 (Figure 6B) induced G2/M-phase cell-growth arrest as well as apoptosis (90).

Researchers developed a new dual inhibitor, compound 80, targeting both PARP and PI3K to treat TNBC. Compound 80 (Figure 6C) showed strong antitumor activity, particularly in BRCA-proficient MDA-MB-468 cells, outperforming existing drugs such as Olaparib and BKM120 in both cell-based and animal models (91).

In a study, a series of new 2-quinolyl-1,3-tropolones derivatives was prepared and tested for antiproliferative activity against several human cancer cell lines. Two compounds showed excellent activity against six cancer cell lines of different tissue origin. The promising compound 81 (Figure 6D) induced apoptotic cell death in ovarian cancer (OVCAR-3, OVCAR-8) and colon cancer (HCT-116) cell lines (92).

A series of novel amide-based PARP-1 inhibitors was designed and synthesized. Among them, compound 82 (Figure 6E) showed strong antiproliferative activity against A549 lung cancer cells (IC_50_ = 2.01 μM) and low toxicity. Compound 82 showed better PARP-1 enzyme inhibition than rucaparib and effectively arrested the cell cycle in S phase and induced apoptosis (93).

Calixarenes, with potential functionalization on the upper and lower rim, have been explored in recent years for the design and construction of anticancer agents. A series of calix arene carbonyl amide derivatives was discovered through optimization of substituted calix (4)arene (CLX-4). The most promising chemical, compound 83 (Figure 6F), identified following cytotoxicity evaluation of the newly created analogs using the MTT test in cancer cell lines, displayed the strongest inhibitory effect against A549 and MDA-MB-231 cells. In addition, the cell inhibition rate against normal HUVEC cells in vitro was only 9.6%, indicating the safety of compound 83. Moreover, compound 83 inhibited migration of MDA-MB-231 cells in a wound-healing assay. Further mechanistic studies indicated that compound 83 could block MDA-MB-231 cell-cycle arrest in the G0/G1 phase by downregulating cyclin D1 and CDK4 and induce apoptosis by upregulating Bax and downregulating Caspase-3, PARP, and Bcl-2 proteins, resulting in reduced DNA synthesis and arrest of cell division (93).

A study describes the design of isoquinolinone and naphthyridinone analogs targeting GLU988 and LYS903 in PARP1. Modifications from a linear propylene linker to a cyclopentene ring improved PK while retaining potency. Further optimization yielded compound 84, a potent PARP1 inhibitor. Compound 84 (Figure 6G) showed strong antitumor activity as a single agent and enhanced the efficacy of chemotherapy drugs such as temozolomide in BRCA1-mutant breast cancer, pancreatic cancer, and Ewing’s sarcoma xenografts (94).

A series of bromophenol-thiosemicarbazone hybrids was designed as PARP-1 inhibitors for anticancer therapy. Among them, compound 85 (Figure 6H) exhibited strong selectivity for PARP-1 over PARP-2 and demonstrated potent anticancer activity against SK-OV-3, Bel-7402, and HepG2 cell lines (95).

A study reports the design, synthesis, and molecular modeling of sixteen conjugates combining ADP with morpholino nucleosides (85) as selective inhibitors of PARP-1, PARP-2, and PARP-3. These compounds mimic the natural substrate NAD (96) (Figure 6I).

A paper describes the synthesis and assessment of XJB-veliparib 87 (Figure 6J), a mitochondria-targeting PARP inhibitor produced by linking veliparib to a pentapeptide mimic that targets mitochondria. Compared with veliparib, XJB-veliparib preserves strong PARP inhibition and protects primary cortical neurons from oxygen-glucose deprivation more effectively. Unlike veliparib, XJB-veliparib prevents mitochondrial NAD loss and PARP formation, preserving mitochondrial structure without impairing nuclear DNA repair (97).

#### 3.4.2. Selective PARP Inhibitors

**Figure 7.**
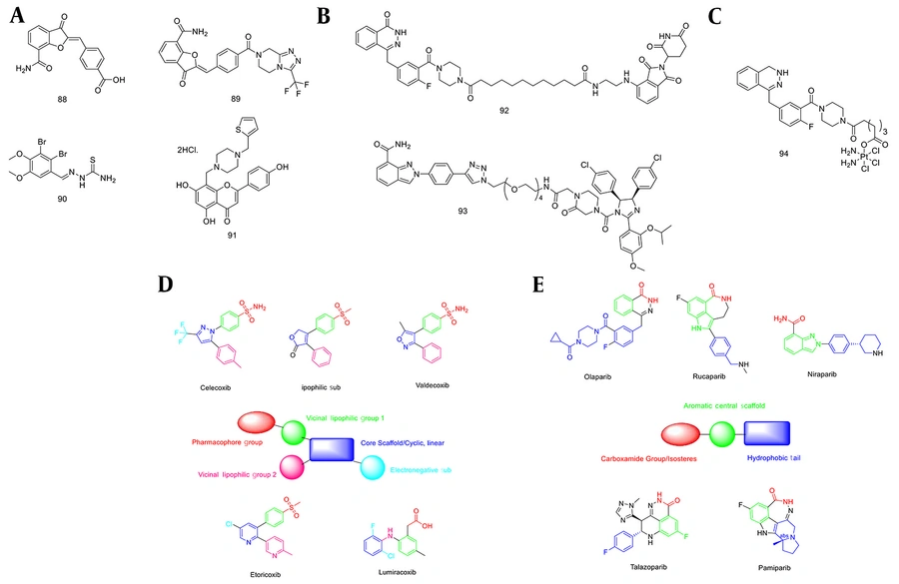
A, Selective PARP-1 inhibitors: tetrazolyl derivatives 88 and 89, DHC-1 90, and amentoflavone analog 91. B, Chemical structures of SK-575 92 and PARP-1 degrader 93, a PARP-1 inhibitor developed using PROTAC technology. C, Chemical structure of platinum analog 94 as a PARP-1 inhibitor. D, Summary of SAR and pharmacophore modeling of PARP-1/COX-2 inhibitors.

Replacement of the tetrazole with a carboxyl group (compound 88, Figure 7A) yielded another promising lead, which was further optimized to produce analogues with PARP-1 inhibitory potencies ranging from 4 to 200 nM. Most compounds were selective for PARP-2. Compound 89 (Figure 7A) emerged as an isoform-selective PARP-1/2 inhibitor and showed selective cytotoxicity against BRCA1-deficient cells versus BRCA1-proficient cells (98).

A study presents DHC-1 (90, Figure 7A), a novel small-molecule compound that selectively inhibits PARP-1. DHC-1 showed strong cytotoxicity against BRCA1-deficient HCC-1937 and BRCA2-deficient Capan-1 cancer cells. It stabilized PARP-1/DNA complexes, inhibited PAR formation, induced DNA double-strand breaks, G2/M phase arrest, and mitochondria-mediated apoptosis. DHC-1 also enhanced the antiproliferative effect of oxaliplatin. In vivo, it effectively suppressed Capan-1 tumor growth (99).

Amentoflavone, a known selective PARP-1 inhibitor, was structurally modified to develop new derivatives. Among the newly developed analogs, compound 91 (Figure 7A) showed strong PARP-1 inhibition and high selectivity over PARP-2. Compound 91 enhanced chemotherapy sensitivity in A549 cells and selectively killed BRCA1-deficient SK-OV-3 cells, making it a promising lead compound for chemosensitizers and BRCA1-deficient cancer therapy (100).

#### 3.4.3. PARP Inhibitors Designed Using PROTAC

A substantial proportion of patients with BRCA1/2 mutations do not respond to PARP inhibitors. This has prompted investigation into the mechanisms underlying PARP inhibitor resistance and approaches to restore sensitivity. Resistance to PARP inhibitors is associated with homologous recombination repair, DNA replication fork stability, PARylation, and epigenetic changes. EZH2, a key epigenetic regulator of transcription through histone methylation, interacts with PARP through mechanisms including DNA homologous recombination, DNA replication, posttranslational modification, and tumor immunity. EZH2 inhibitors are transitioning from laboratory research to clinical application; however, combination strategies in cancer therapy remain inadequately investigated. A novel pharmacological design integrating PARP inhibitors and EZH2 inhibitors with proteolysis targeting chimera (PROTAC) approaches has been proposed as a solution to PARP inhibitor resistance (101).

Researchers developed PARP1 degraders using the PROTAC strategy, leading to the discovery of SK-575 (compound 92, Figure 7B). SK-575 significantly disables PARP-1 and suppresses cancer cell proliferation, particularly in cells with BRCA1/2 mutations, even at low picomolar levels. In mouse models, SK-575 demonstrated substantial tumor suppression both as a single agent and in combination with chemotherapeutic medicines such as temozolomide and cisplatin, suggesting its potential as an effective and long-lasting cancer treatment (102).

A study focused on developing a small-molecule PARP-1 degrader using the PROTAC strategy. The representative compound 93 (Figure 7B) effectively induces PARP-1 cleavage and triggers programmed cell death in MDA-MB-231 breast cancer cells (103).

#### 3.4.4. Organometallic Compounds

Platinum-based drugs have long served as the cornerstone of first-line chemotherapy for a wide range of solid tumors. Their clinical success has driven extensive research into the biological activity of DNA-binding metal complexes as a promising class of therapeutic agents. Advances in the molecular biology of cancer have paved the way for combining DDR inhibitors, such as PARP inhibitors, to achieve synergistic cancer cell killing (104). In one study, nine novel platinum (IV) complexes modified with Olaparib pharmacophores were synthesized to overcome cisplatin (CDDP) resistance and reduce side effects. The lead compound, complex 94 (Figure 7C), showed strong PARP-1 inhibition and superior anticancer activity against both parental and CDDP-resistant TNBC cells. Mechanistic studies demonstrated that 94 enhanced intracellular accumulation, reversed CDDP resistance by inhibiting DNA repair, increased DNA damage, and activated mitochondria-dependent apoptosis (105).

#### 3.4.5. COX-2/PARP-1 Dual Inhibitors

**Figure 8.**
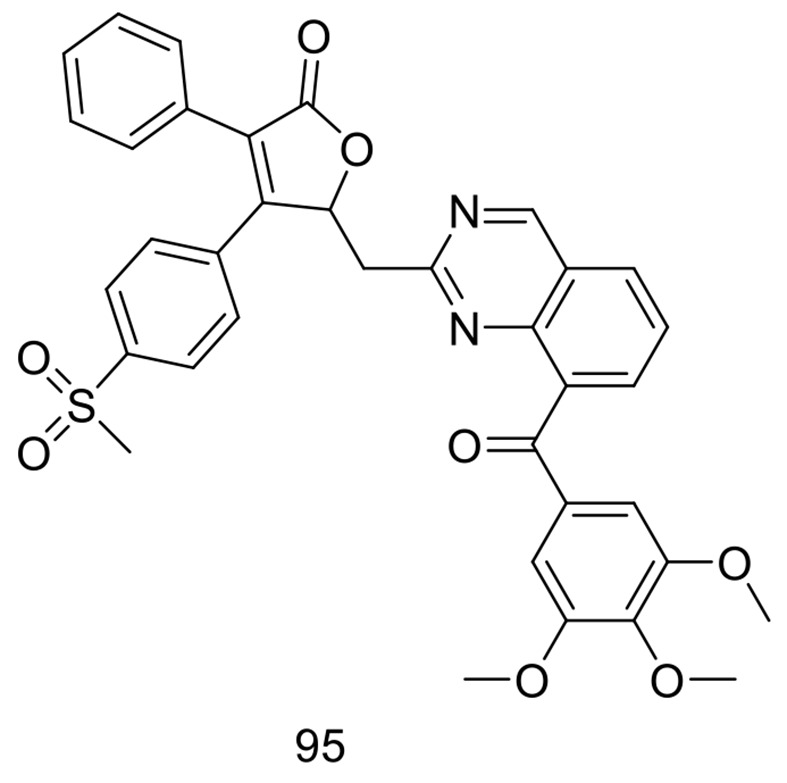
Chemical structure of designed compound 95 as a potential dual-acting COX-2/PARP-1 inhibitor.

COX-2/PARP-1 dual inhibitors are an experimental cancer treatment approach that targets COX-2 and PARP-1 simultaneously. COX-2 inhibitors are renowned for their anti-inflammatory and anti-angiogenic properties, whereas PARP-1 inhibitors are used to prevent DNA repair, particularly in cancer cells harboring BRCA mutations. Dual inhibitors that combine these mechanisms could provide a stronger and more targeted approach to cancer treatment. Integrating COX-2 and PARP-1 suppression may provide broader antitumor efficacy by targeting both tumor growth and DNA repair processes. Some cancers develop resistance to single-agent PARP inhibitors; dual inhibition may overcome this resistance. Dual inhibitors targeting both COX-2 and PARP-1 may therefore result in a more substantial reduction in tumor development and metastasis. In this regard, considering all chemical structures disclosed in this manuscript, compound 95 was designed as a potential COX-2/PARP-1 dual inhibitor (Figure 8).

**Figure 9.**
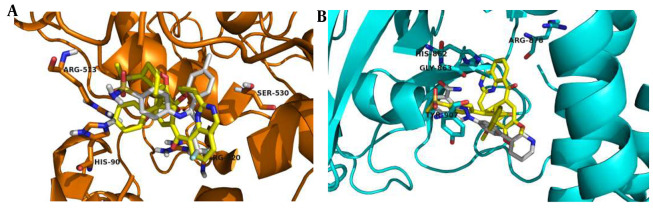
Binding mode of designed compound 95, shown in yellow, as a potential COX-2/PARP-1 dual inhibitor within the active sites of COX-2 and PARP-1. (A) Binding conformation of compound 95 within the active site of COX-2, compared with bound celecoxib (PDB ID: 1CX2; gray), highlighting key interactions within the catalytic pocket. (B) Docking configuration of compound 95 within the active site of PARP-1 bound to its native ligand (PDB ID: 7KK5), highlighting key binding interactions.

**Figure 10.**
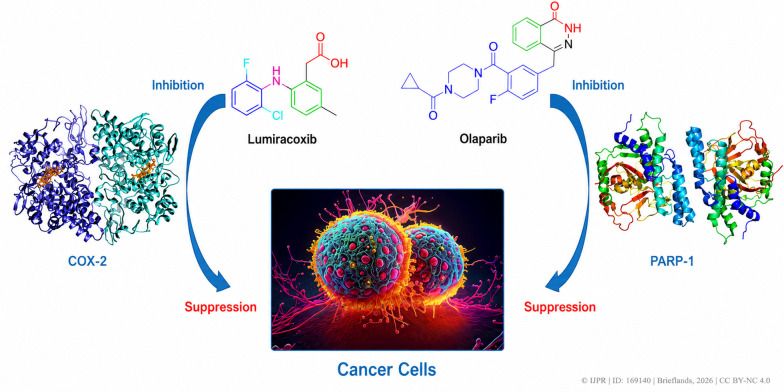
Graphical abstract

Compound 95 was docked with the two aforementioned enzymes, COX-2 (PDB ID: 1CX2) (106) and PARP-1 (PDB ID: 7KK5) (107), using the VinaDock® docking tool software (108). As shown, the compound is well positioned in the active sites of COX-2 and PARP-1 (Figure 9). The binding energies of celecoxib and compound 95 with the COX-2 enzyme were -10.6 and -8.9 kcal/mol, respectively. In docking with the PARP enzyme, the binding energies of the native ligand and compound 95 were -11.0 and -10.5 kcal/mol, respectively. The graphical abstract is provided in Figure 10.

## 4. Conclusions

The use of PARP-1/COX-2 dual inhibitors represents a viable therapeutic approach targeting two critical and interconnected pathways involved in cancer development and progression: DNA repair and inflammatory responses. These dual-action agents inhibit PARP-1, a key enzyme in the base excision repair pathway, and COX-2, a major mediator of inflammation and tumor-promoting prostaglandins, thereby offering the potential to enhance anticancer efficacy, reduce resistance, and lessen the need for combination therapies. Preclinical studies have demonstrated synergistic effects, including increased DNA damage, reduced cell viability, impaired angiogenesis, and modulation of the tumor microenvironment. Consequently, PARP-1/COX-2 dual inhibitors may be particularly beneficial in tumors with pronounced inflammatory features or deficiencies in gene repair systems. The present analysis provided concise SAR statistics for COX-2 and PARP1 inhibitors derived from recently published literature. The development of selective COX-2 inhibitor drugs focuses on specific structural differences between COX-1 and COX-2. COX-2 has a more constricted and adaptable hydrophobic adjacent pocket due to a valine residue at position 523, whereas COX-1 contains a more restrictive isoleucine. The presence of a polar functional group, typically a sulfonamide or methylsulfonyl group, that occupies the side cavity and forms hydrogen bonds with key residues such as Arg513 and His90 is the primary determinant of COX-2 selectivity. Celecoxib serves as a prototype because of its pyrazole scaffold and pharmacophore-type sulfonamide moiety. SAR assessment indicated that the electronic properties of substituents on the aromatic rings strongly influence ligand binding affinity. Electron-withdrawing substituents, such as halogens, were found to improve interactions with the active site and increase metabolic stability.

Compounds targeting the PARP superfamily, particularly selective PARP-1 inhibitors, mimic the nicotinamide moiety of nicotinamide adenine dinucleotide (NAD), the natural ligand of PARP-1. Early SAR studies used simple benzamide compounds and yielded moderate inhibition. Subsequent optimization has focused on developing more potent scaffolds, including phthalazinone and tricyclic fused-ring frameworks. Olaparib contains a phthalazinone core that enables π stacking and hydrogen bonding through interactions with residues such as Tyr907 and His862. Beyond catalytic inhibition, some PARP-1 inhibitors also immobilize the enzyme on DNA, thereby enhancing cytotoxicity in neoplastic cells. DNA trapping is influenced by molecular rigidity and lipophilicity. Talazoparib exhibits strong DNA-trapping capability attributable to its rigid tricyclic core and optimally designed side chains. SAR research has evaluated the effects of linker size and flexibility on dual binding to PARP-1 and DNA. These findings indicate that polycyclic structures can be leveraged to develop agents that simultaneously inhibit the aforementioned enzymes. Studies have also shown that polycyclic scaffolds may inhibit additional cancer-promoting enzymes, such as topoisomerase-2 (TPO-2). Currently, the most common PARP-1 inhibitors have structures similar to the core scaffold of non-selective COX inhibitors or NSAIDs (Figure 7D).

In brief, these data suggest that combining NAD-mimetic structures, such as phthalazinone, with selective COX-2 inhibitor motifs, such as diaryl heterocycles containing the sulfonamide pharmacophore and its isosteres, may provide a promising framework for the continued development of dual-enzyme inhibitors. Further research is needed to develop pharmaceuticals that inhibit both PARP-1 and COX-2.

In summary, to provide a more comprehensive review article on the design and synthesis of novel chemical compounds as COX-2/PARP-1 dual inhibitors, additional compounds must be generated. Moreover, further clinical development and evaluation are required to improve their pharmacological properties and confirm their safety and efficacy in cancer patients. The graphical absract of this study has been provided in Figure 10.
